# Effects of repetitive transcranial magnetic stimulation combined with repetitive peripheral magnetic stimulation on upper limb motor function after stroke: a systematic review and meta-analysis

**DOI:** 10.3389/fneur.2024.1472837

**Published:** 2024-11-12

**Authors:** Shanshan Luo, Zhu Wen, Ying Liu, Tao Sun, Li Xu, Qian Yu

**Affiliations:** ^1^Hospital of Chengdu University of Traditional Chinese Medicine, Chengdu, China; ^2^School of Clinical Medicine, Southwest Medical University, Luzhou, China; ^3^Department of Rehabilitation Medicine, Sichuan Provincial People's Hospital, School of Medicine, University of Electronic Science and Technology of China, Chengdu, China

**Keywords:** motor function, repetitive transcranial magnetic stimulation (rTMS), repetitive peripheral magnetic stimulation (rPMS), stroke, meta-analysis, randomized controlled trial

## Abstract

**Objective:**

To evaluate the effectiveness of repetitive transcranial magnetic stimulation (rTMS) combined with repetitive peripheral magnetic stimulation (rPMS) on upper limb motor dysfunction after stroke.

**Methods:**

We systematically searched databases up to May 2024, including PubMed, Embase, Cochrane Library, Web of Science, CNKI, VIP, Wanfang, and CBM. Randomized controlled trials (RCTs) examining the application of rTMS combined rPMS on upper limb motor dysfunction after stroke were included based on predefined inclusion criteria. We used Cochrane Risk of Bias 2 tool to assess bias risk of the included RCTs. Meta-analysis was conducted using RevMan 5.4 and Stata 17.0 software.

**Results:**

A total of 9 RCTs involving 483 participants were included in this study. Compared with the control groups that used either conventional therapy or rTMS alone, the experimental group that used rTMS combined rPMS showed significant improvements in stroke patients' upper limb motor function [MD = 3.65, 95% CI (2.75, 4.54), *P* < 0.05], ability of daily living [MD = 4.50, 95% CI (3.50, 5.50), *P* < 0.05], and spasticity [MD = –0.34, 95% CI (−0.48, −0.20), *P* < 0.05]. Meanwhile, in terms of neurophysiological indicators, significant differences were found both for motor evoked potential latency [MD = −1.77, 95% CI (−3.19, −0.35), *P* < 0.05] and motor evoked potential amplitude [MD = 0.25, 95% CI (0.01, 0.49), *P* < 0.05].

**Conclusion:**

This study provides low-level evidence that the therapy of LF-rTMS or HF-rTMS combined with rPMS can improve the upper limb motor function and daily living ability of stroke patients. However, given that the low quality of the evidence for the evaluation results, further evidence from high-quality studies is needed to substantiate this conclusion.

**Systematic review registration:**

https://www.crd.york.ac.uk/prospero/display_record.php?ID=CRD42024539195, PROSPERO Platform [CRD42024539195].

## 1 Introduction

Stroke often results in varying degrees and types of functional impairments. Over the past two decades, although advancements in medical science have reduced stroke mortality, the incidence and number of stroke survivors are still increasing, and stroke remains a leading cause of morbidity and disability worldwide (1–3). Despite the early application of interventions and treatments, 75% of stroke patients experience upper limb motor dysfunction (4), posing significant challenges for recovery. This dysfunction is closely associated with decreased daily living activities and a deteriorating quality of life (5, 6), imposing a substantial economic burden on families and society. Therefore, exploring effective methods to promote motor function recovery and improve prognosis post-stroke has become a primary focus of neurorehabilitation practitioners.

One key aspect of post-stroke motor recovery is neuroplasticity, which may occur spontaneously after stroke or be facilitated by appropriate rehabilitation interventions like non-invasive brain stimulation techniques (7–9). Repetitive transcranial magnetic stimulation (rTMS), as a non-invasive brain stimulation (NIBS) technique, can promote the recovery of motor function after stroke by directly acting on the cerebral hemisphere, modulating the excitability of the cerebral cortex, participating in and inducing the neuroplasticity changes after stroke (10–12).

Repetitive peripheral magnetic stimulation (rPMS) is another non-invasive technique that targets peripheral nerves and muscles outside the brain. By applying specific magnetic stimulation to peripheral tissues, rPMS painlessly excites nerves or muscles, causing contractions in paralyzed limbs, which can enhance proprioceptive input from the affected limbs and indirectly modulate the excitability of the cerebral cortex, thereby promoting limb function recovery (13–15), particularly in patients with severe upper limb dysfunction post-stroke (16).

Recent meta-analyses and reviews have confirmed the efficacy of rTMS or rPMS alone in improving upper limb motor function in stroke patients (12, 17–22). However, evidence supporting their combined synergistic effect is lacking. Therefore, the primary objective of this study is to conduct a systematic review and meta-analysis of recently published randomized controlled trials to evaluate the combined effect of rTMS and rPMS on upper limb motor function in post-stroke patients, in comparison with rTMS alone, rPMS alone, or conventional therapy.

## 2 Methods

### 2.1 Search strategy

This study is reported following the PRISMA (Preferred Reporting Items for Systematic Reviews and Meta-Analyses) guidelines (23) and has been registered on the PROSPERO Platform (CRD42024539195). The databases PubMed, Embase, Cochrane Library, Web of Science, CNKI, VIP, Wanfang, and CBM were systematically searched for studies published up to May 2024, and the search language was limited to Chinese and English. The search strategies for this study includes keywords such as “stroke,” “cerebrovascular accident,” “upper extremity,” “repetitive transcranial magnetic stimulation,” and “repetitive peripheral magnetic stimulation.” For more detailed information of the search strategies, please refer to Supplementary material.

### 2.2 Inclusion criteria and study selection

Following the PICOS framework, the inclusion criteria were: I: Participants: patients experiencing their first stroke, including cerebral infarction or hemorrhage. II: Intervention: interventions using combined rTMS and rPMS treatments. III: Comparison: interventions using conventional therapy, and interventions using rTMS or rPMS isolated. IV: Outcome Measures: upper limb motor function outcomes. V: Study Design: randomized controlled trials.

Two authors (SL and ZW) independently screened the literature. The retrieved literature records were imported into Endnote 20. After removing duplicates, titles and abstracts were preliminarily assessed according to the inclusion criteria to identify relevant studies. Conference abstracts, case reports, non-randomized trials, reviews, conference papers, and dissertations were excluded. Full-text reading was conducted to confirm eligibility, and eligible randomized controlled trials were included in the systematic review.

### 2.3 Data extraction

Two researchers (SL and ZW) independently reviewed and extracted the following data from each study: author, publication year, sample size, gender, age, disease type, disease course, interventions, and outcome measures. Disagreements were resolved through discussion, and if there is a discrepancy, a third researcher (QY) is consulted.

### 2.4 Risk of bias assessment

The Cochrane RoB 2 tool was used to assess the risk of bias in the included studies. The overall risk of bias for each study was determined based on the following aspects such as randomization process, differences from the intended interventions, presence of missing outcome data, outcome measurement, and selective reporting of results. Each study was then classified as “high risk”, “some concerns”, or “low risk” based on the overall bias risk. A final cross-check was performed, and any discrepancies during the assessment were resolved by a third reviewer. To provide a more comprehensive evaluation of the methodological quality of the included studies, the Pedro scale was also used (24). The Pedro scale evaluates various aspects of each study, including the use of blinding, the randomization process, the reporting of baseline characteristics, point estimates, and variability measurements, as well as data analysis (intent-to-treat analysis) and the adequacy of follow-up. It serves as a supplement to the Cochrane RoB 2 tool.

### 2.5 Quality of evidence rating

The quality of evidence provided by this meta-analysis was evaluated using the Grading of Recommendations, Assessment, Development and Evaluations (GRADE) framework (25). This evaluation considered the risk of bias, inconsistency of results, indirectness of evidence, imprecision, and publication bias.

### 2.6 Data synthesis and analysis

Statistical analysis was conducted using RevMan 5.4. Since the outcome measures of the included studies were continuous variables, the mean difference (MD) was used to represent the effect size and the 95% confidence interval (CI) was calculated, and the difference was considered statistically significant at *P* < 0.05. The *I*^2^ statistic was used to evaluate the degree of heterogeneity among the studies, and *I*^2^ statistical values between 25% and 50% may indicate low heterogeneity, *I*^2^ values between 50% and 75% may indicate moderate heterogeneity, and *I*^2^ values greater than 75% may indicate high heterogeneity (26). If *I*^2^ ≥ 50%, a random effects model was employed to assess the pooled effect size. Otherwise, a fixed effects model was employed. As the purpose of this study was to evaluate the synergistic effect of rTMS combined with rPMS, any group receiving combination therapy of rTMS and rPMS will be considered as the experimental group. If an article included multiple control groups (rTMS alone, rPMS alone, or conventional treatment), they were treated as separate trials to provide a more in-depth review of the combined effect. If the results of the included studies did not provide mean values and standard deviations but were reported as medians and interquartile ranges (IQR), appropriate statistical methods (27) were employed to convert the medians and IQRs to means and standard deviations.

Meta-regression analysis was performed using Stata 17.0 software to assess the impact of clinical characteristics (such as gender, age) on the meta-analysis results. Furthermore, sensitivity analysis and Egger's test were employed to evaluate the stability of the results and to check for publication bias.

## 3 Results

### 3.1 Search results

**Figure 1:**
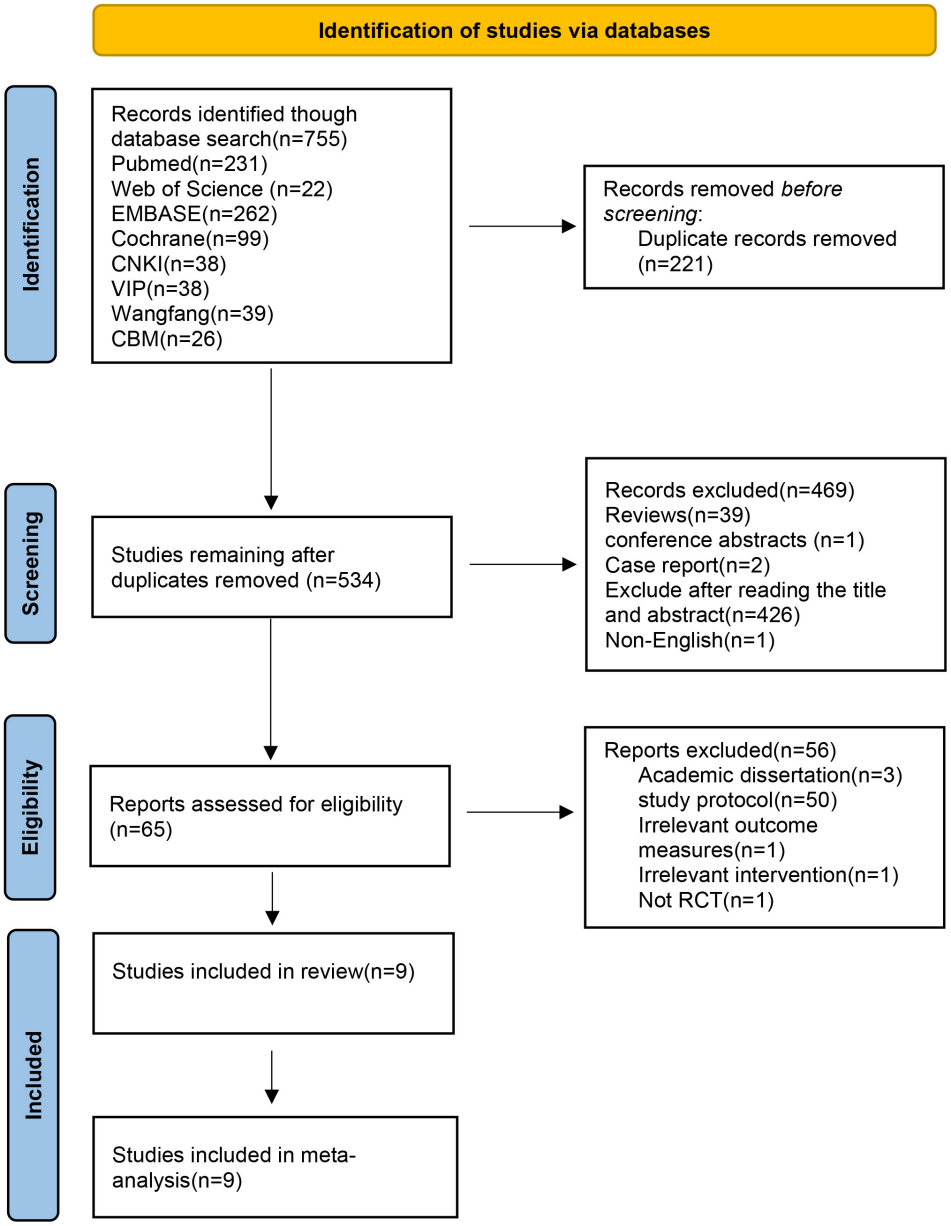
Flowchart of literature screening.

A total of 755 articles were retrieved from databases. After removing duplicates, the remaining 534 articles were preliminarily screened based on their titles and abstracts. The eligibility of 65 articles was thoroughly assessed by full-text reading, and finally, nine articles were included in the study. The screening process is shown in Figure 1.

### 3.2 Characteristics of included studies

This study included nine articles (28–36) with a total of 483 participants. Among them, five studies (29, 30, 33, 34, 36) included two control groups, and one study (31) included three control groups, and were therefore divided into two or three independent trials. Consequently, a total of 16 trials were included in the meta-analysis. Tables 1, 2 presented the demographic characteristics of participants and the stimulation protocols used in each study. All participants were older than 18 years, and the course of disease in most patients was within 6 months of the onset. In terms of stroke type, ischemic (73.14%) was more common than hemorrhagic (26.86%). All studies used rTMS combined with rPMS as the intervention for the experimental group, among them, five articles (28–31, 34) mentioned the order of application of the two magnetic stimulations. Regarding rTMS protocols, three studies (30, 32, 35) used low-frequency rTMS (LF-rTMS), four studies (29, 31, 33, 34) used high-frequency rTMS (HF-rTMS), and two studies (28, 36) employed intermittent theta burst stimulation (iTBS). In terms of the magnetic stimulation devices, three studies (31, 34, 36) used the YRD CYY-I, two (29, 35) used the YRD CCY-II, two (28, 33) used the MagVenture-MagProX100, and the remaining two studies (30, 32) used Magstim and Magventure-MagPro30, respectively. For the magnetic coils, three studies (28, 30, 36) used figure-eight coils, one study (34) used a circular coil, one study (29) used a double-ended circular coil, and one study (31) used both a circular coil and a figure-eight coil. The remaining three studies (32, 33, 35) did not report the type of coil used. As for the rPMS protocols, most studies (29–35) used high-frequency stimulation (frequency range from 5 to 20 Hz), and two studies (28, 36) used peripheral iTBS. The stimulation points of rPMS included the Erb's point of the affected upper limb, cervical nerve root, radial nerve, and paralyzed muscle groups on the affected side. Regarding the number of treatment sessions, most studies conducted one session per day, 5 days per week. The total number of intervention sessions ranged from 10 to 40. Specific details of the characteristics of each study are shown in Tables 1, 2.

**Table 1:** Characteristics of studies included in the meta-analysis.

**References**	**Sample size**	**Intervention**	**Sex (M/F)**	**Age [(*x* ±*s*), years]**	**Type of stroke (ischemic/ hemorrhagic)**	**Course of disease (*x* ±*s*)**	**Outcome measures**
Chang et al. (28)	• I: 14 • C: 14	• I: iTBS + rPMS • C: iTBS + shamp rPMS	• I: 4/10 • C: 11/3	• I: 51.4 ± 12.1 • C: 55.6 ± 10.3	• I: 6/8 • C: 4/10	• Not Reported • Not Reported	FMA-UE, ARAT, FIM-selfcare, SIS
Chen et al. (35)	• I:22 • C:20	• I: LF-rTMS + rPMS • C: LF-rTMS	• I: 16/6 • C: 14/6	• I: 62.18 ± 10.24 • C: 59.55 ± 8.86	• I:22/0 • C:20/0	• I: 7.23 ± 0.66 d • C: 8.93 ± 4.01 d	FMA-UE, MBI
Liang et al. (29)	• I:15 • C_1_:15 • C_2_:15	• I: HF-rTMS + rPMS • C_1_: HF-rTMS • C_2_: Conventional Therapy + sham rTMS	• I: 10/5 • C_1_: 11/4 • C_2_: 10/5	• I: 56.6 ± 9.1 • C_1_: 54.1 ± 9.6 • C_2_: 54.3 ± 7.6	• I: 5/10 • C_1_: 8/7 • C_2_: 8/7	• I: 2.3 ± 1.8 m • C_1_: 2.3 ± 1.5 m • C_2_: 2.5 ± 1.4 m	FMA-UE, BI, FCA, RMT, MEP amplitude, MEP latency SICI
Meng et al. (36)	• I:15 • C_1_:15 • C_2_:15	• I: iTBS + rPMS • C_1_: iTBS + shamp rPMS • C_2_: Conventional therapy	• I: 8/7 • C_1_: 7/8 • C_2_: 8/7	• I: 55.3 ± 7.5 • C_1_: 52.5 ± 13.5 • C_2_: 53.9 ± 10.7	• I: 6/9 • C_1_: 7/8 • C_2_: 8/7	• I: 36.1 ± 8.2 d • C_1_: 34.7 ± 7.9 d • C_2_: 33.6 ± 6.8 d	FMA-UE, MBI, MEP amplitude, MEP latency
Qin et al. (30)	• I:20 • C_1_:15 • C_2_:14	• I: LF-rTMS + rPMS • C_1_: LF-rTMS • C_2_: Conventional Therapy	• I: 11/9 • C_1_: 9/6 • C_2_: 11/3	• I: 60.05 ± 9.97 • C_1_: 55.87 ± 10.5 • C_2_: 59.43 ± 9.12	• I: 20/0 • C_1_: 15/0 • C_2_: 14/0	• I: 3.45 ± 1.76 m • C_1_: 3.20 ± 1.93 m • C_2_: 2.85 ± 1.74 m	FMA-UE, MBI, MAS, ALFF
Wu et al. (31)	• I:15 • C_1_:15 • C_2_:15 • C_3_:15	• I: HF-rTMS + rPMS • C_1_: HF-rTMS + shamp rPMS • C_2_: shamp rTMS + rPMS • C_3_: Conventional Therapy + sham rTMS + shamp rPMS	• I: 10/5 • C_1_: 12/3 • C_2_: 11/4 • C_3_: 15/0	• I: 54.60 ± 11.16 • C_1_: 57.00 ± 10.76 • C_2_: 54.87 ± 11.6 • C_3_: 55.33 ± 10.3	• I: 8/7 • C_1_: 5/10 • C_2_: 6/9 • C_3_: 8/7	• I: 38 (28, 50)^a^ d • C_1_: 34 (20, 46)^a^ d • C_2_: 26 (21, 59)^a^ d • C_3_: 32 (21, 72)^a^ d	FMA-UE, MBI, WMFT
Xia et al. (33)	• I: 40 • C_1_: 40 • C_2_: 40	• I: HF-rTMS + rPMS • C_1_: HF-rTMS • C_2_: Conventional therapy	• I: 24/16 • C_1_: 25/15 • C_2_: 22/18	• I: 70.1 ± 1.7 • C_1_: 69.3 ± 1.9 • C_2_: 69.9 ± 1.8	• I: 40/0 • C_1_:4 0/0 • C_2_: 40/0	• I: 79.1 ± 37.6 d • C_1_: 76.6 ± 39.3 d • C_2_: 72.3 ± 34.8 d	FMA-UE, MBI, MAS, CSI, CMCT MEP latency
Yan et al. (32)	• I: 18 • C: 17	• I: LF-rTMS + rPMS • C: LF-rTMS + shamp rPMS	• I: 14/4 • C: 12/5	• I: 60.56 ± 8.75 • C: 58.29 ± 17.25	• I: 14/4 • C: 10/7	• I: 20.0 (16.0, 75.0)^b^ d • C: 30.0 (16.0, 97.5)^b^ d	FMA-UE, MBI, NIHSS
Zhang et al. (34)	• I: 20 • C_1_: 20 • C_2_: 20	• I: HF-rTMS + rPMS • C_1_: HF-rTMS • C_2_: Conventional therapy	• I: 17/3 • C_1_: 16/4 • C_2_: 16/4	• I: 55.5 ± 10.7 • C_1_: 57.9 ± 9.3 • C_2_: 55.6 ± 12.4	• I: 15/5 • C_1_: 15/5 • C_2_: 10/10	• I: 55.2 ± 46.9 d • C_1_: 55.5 ± 42.6 d • C_2_: 52.9 ± 35.3 d	FMA-UE, MBI, RMT, RMS

I, intervention group; C, control group; M, male; F, female; *x* ± *s*, mean ± SD; m, month; d, day.

^a^Mean (Q25, Q75).

^b^Mean (Xmin, Xmax).

ARAT, Action Research Arm Test; FCA, Comprehensive Functional Assessment; FMA-UE, Fugl-Meyer Assessment-Upper Extremity; FIM-Selfcare, self-care domain of the Functional Independence Measure; SIS, Stroke impact Scale; HF-rTMS, High Frequency-repetitive Transcranial Magnetic Stimulation; LF-rTMS, Low Frequency-repetitive Transcranial Magnetic Stimulation; iTBS, intermittent theta-burst stimulation; MAS, Modified Ashworth scale; BI, Barthel Index; MBI, Modified Barthel Index; NIHSS, National Institutes of Health Stroke Scale; WMFT, Wolf Motor Function Test; CSI, clinic spasticit index; CMCT, central motion conduction time; RMS, root mean square; RMT, resting motor threshold; SICI, short-interval intracortical inhibition.

**Table 2:** Details of the magnetic stimulation protocol in the included studies.

**References**	**Type of machine**	**Type of coil**	**Intervention of experimental group**	**Application order; ISI**	**Stimulation site**	**Intensity, %RMT**	**Fre (Hz); pulses per session**	**Duration per treatment; total treatment course**
Chang et al. (28)	MagVenture-MagProX100	Figure-of-eight coil	iTBS + rPMS	First rPMS, then rTMS; NR	• rTMS: M1 of the affected side • rPMS: the radial nerve of the affected limb	• rTMS: 70 • rPMS: NA	• rTMS: a; 600 • rPMS: a; 600	• rTMS: 200 s; 2/ d, 5 d/w, 2 w • rPMS: 200 s; 2/ d, 5 d/w, 2 w
Chen et al. (35)	YRD CCY-II	NR	LF-rTMS + rPMS	NR; NR	• rTMS: M1 of the unaffected side • rPMS:	• rTMS: 110 • rPMS: NA	• rTMS: 1; 1,200 • rPMS: 20; 400	• rTMS: NR; daily, 4 w • rPMS: NR; daily, 4 w
Liang et al. (29)	YRD CCY-II	Double-ended circular coil	HF-rTMS + rPMS	Each peripheral stimulation was delivered 20 ms after rTMS; 20 ms	• rTMS: M1 of the affected side • rPMS: the seventh cervical nerve root	• rTMS: 80 • rPMS: NR	• rTMS: 5; 1,200 • rPMS: 5; 1,200	• rTMS: 20 min; daily, 5 d/w, 4 w • rPMS: 20 min; daily, 5 d/w, 4 w
Meng et al. (36)	YRD CCY-1	Figure-of-eight coil	iTBS + rPMS	NR; NR	• rTMS: M1 of the affected side • rPMS:	• rTMS: NR • rPMS: NR	• rTMS: a; 600 • rPMS: a; 600	• rTMS: 3 min; daily, 5 d/w, 2 w • rPMS: 3 min; daily, 5 d/w, 2 w
Qin et al. (30)	Magstim	Figure-of-eight coil	LF-rTMS + rPMS	First rTMS, then rPMS; NR	• rTMS: M1 of the unaffected side • rPMS:	• rTMS: 90 • rPMS: NR	• rTMS: 1; 1,200 • rPMS: 10; 1,200	• rTMS: NR; daily, 5 d/w, 8 w • rPMS: NR; daily, 5 d/w, 8 w
Wu et al. (31)	YRD CCY-I	• rTMS: Figure-of-eight coil • rPMS: Circular coil	HF-rTMS + rPMS	First rTMS, then rPMS; NR	• rTMS: M1 of the affected side • rPMS: the cervical nerve root	• rTMS: 80 • rPMS: NA	• rTMS: 10; 1,000 • rPMS: 10; 1,000	• rTMS: NR; daily, 5 d/w, 3 w • rPMS: NR; daily, 5 d/w, 3 w
Xia et al. (33)	MagVenture-MagProX100	NR	HF-rTMS + rPMS	NR; NR	• rTMS: M1 of the affected side • rPMS: Nerves of the affected limb	• rTMS: 90 • rPMS: +5	• rTMS: 20; 140 • rPMS: 8; 300	• rTMS: NR; daily, 5 d/w, 4 w • rPMS: NR; daily, 5 d/w, 4 w
Yan et al. (32)	MagVenture-MagPro30	NR	LF-rTMS + rPMS	NR; NR	• rTMS: M1 of the unaffected side • rPMS: muscles of the affected limb	• rTMS: 80 • rPMS: NA	• rTMS: 1; 1,200 • rPMS: 10; 1,200	• rTMS: 20 min; daily, 5 d/w, 2 w • rPMS:NR; daily, 5 d/w, 2 w
Zhang et al. (34)	YRD CYY-I	Circular coil	HF-rTMS + rPMS	First rTMS, then rPMS; NR	• rTMS: M1 of the affected side • rPMS:	• rTMS: 80 • rPMS:150	• rTMS: 10; NR • rPMS: 10; NR	• rTMS: 17.15 min; daily, 5 d/w, 2 w • rPMS: 7.15 min; daily, 5 d/w, 2 w

ISI, interstimulus interval; Fre, frequency; RMT, resting motor threshold; , around the acromial end of the clavicle; , around the abdominal muscles of the triceps; , around the dorsal extensor muscles of the wrist; , each peripheral stimulation was delivered 20 ms after rTMS; , the Erb's point of the affected upper limb; min, minute(s); a, 50 Hz within the plexus, 5 Hz between the plexus; d, day(s); s, second; w, week(s); M1, the primary motor cortex; NR, not reported; NA, not applicable.

### 3.3 Quality assessment of studies

**Figure 2:**
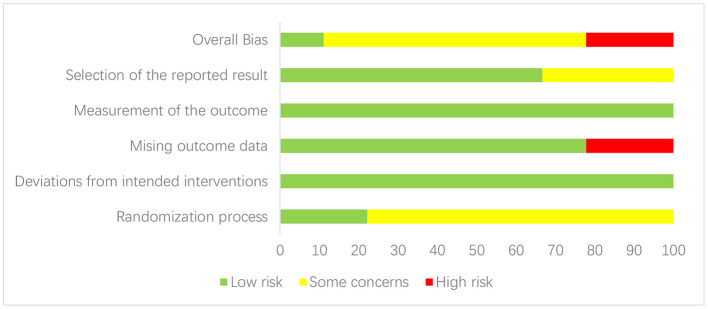
Risk of bias summary.

**Figure 3:**
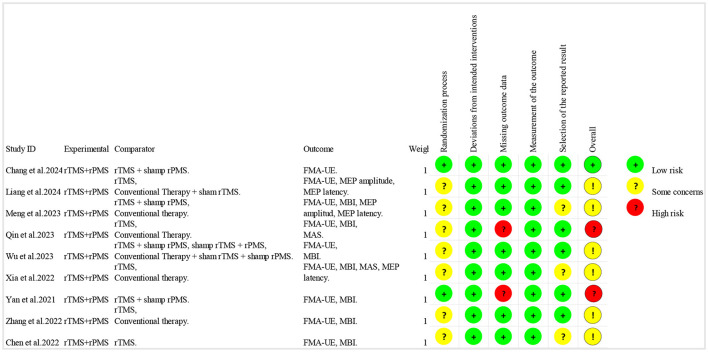
Risk of bias graph.

The Cochrane RoB 2 tool was used to assess the risk of bias of the included studies. Due to the lack of mention of randomization methods during the randomization process or the possibility of potential selective reporting, six studies (29, 31, 33–36) were rated as “some concerns.” Due to missing outcome data, two studies (30, 32) were rated as “high risk,” and the remaining study (28) was rated as “low risk.” Meanwhile, in terms of the PEDro scale scores, the average PEDro score of the included studies was 7 points (range 5–10). The detailed information on the bias risk of each study is shown in Figures 2, 3 and Table 3.

**Table 3:** PEDro scale scores for the included studies.

**References**	**Eligibility criteria**	**Random allocation**	**Concealed allocation**	**Baseline comparability**	**Blind subjects**	**Blind therapists**	**Blind assessors**	**Adequate follow-up**	**Intention-to-treat analysis**	**Between-group comparisons**	**Point estimates and variability**	**Total score (0–10)**
Chang et al. (28)	Y	1	1	1	1	1	1	1	1	1	1	10
Chen et al. (35)	Y	1	0	1	0	0	0	1	1	1	1	6
Liang et al. (29)	Y	1	0	1	1	0	1	1	1	1	1	8
Meng et al. (36)	Y	1	0	1	1	1	1	1	1	1	1	9
Qin et al. (30)	Y	1	0	1	0	0	0	0	1	1	1	5
Wu et al. (31)	Y	1	0	1	1	1	1	1	1	1	1	9
Xia et al. (33)	Y	1	0	1	0	0	0	1	1	1	1	6
Yan et al. (32)	Y	1	1	1	1	0	1	1	1	1	1	9
Zhang et al. (34)	Y	1	0	1	0	0	1	1	1	1	1	7

### 3.4 Meta-data analysis

#### 3.4.1 Primary outcomes

**Figure 4:**
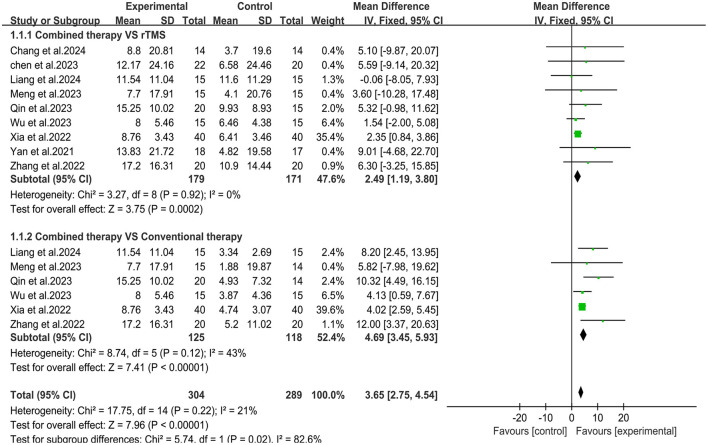
Forest plot of Fugl-Meyer Assessment-Upper Extremity.

Nine studies (28–36) used the Fugl-Meyer Assessment-Upper Extremity (FMA-UE) scale to assess upper limb motor function after stroke (Figure 4). Meta-analysis showed that the overall mean difference in upper limb Fugl-Meyer assessment was MD = 3.65, 95% CI (2.75, 4.54), *P* < 0.05. According to the fixed effects model, compared with the rTMS group, the upper limb motor function scores in the combined group were significantly improved [MD = 2.49, 95% CI (1.19, 3.80), *P* < 0.05, *I*^2^ = 0%]. Compared with the conventional treatment group, the upper limb motor function in the combined group also improved significantly, but the results showed mild heterogeneity [MD = 4.69, 95% CI (3.45, 5.93), *P* < 0.05, *I*^2^ = 43%].

**Figure 5:**
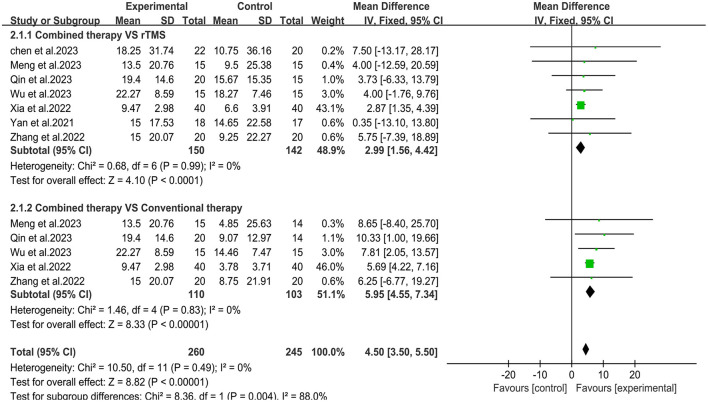
Forest plot of Modified Barthel Index.

Seven studies (30–36) used the Modified Barthel Index (MBI) scale to assess changes in daily living ability before and after treatmen (Figure 5). Meta-analysis showed that the overall mean difference was MD = 4.50, 95% CI (3.50, 5.50), *P* < 0.05. According to the fixed effects model, compared with the rTMS group, the daily living activity scores in the combined group were significantly improved [MD = 2.99, 95% CI (1.56, 4.42), *P* < 0.05, *I*^2^ = 0%]. Compared with the conventional treatment group, the upper limb daily living activity in the combined group also improved significantly [MD = 5.95, 95% CI (4.55, 7.34), *P* < 0.05, *I*^2^ = 0%].

#### 3.4.2 Secondary outcomes

**Figure 6:**
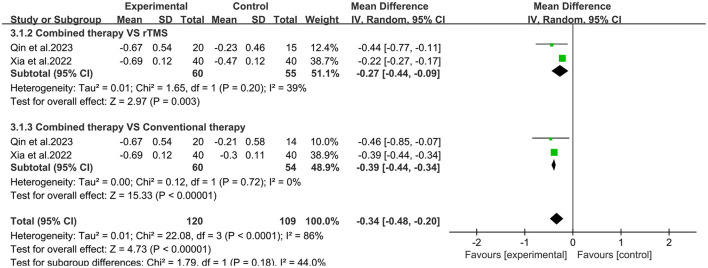
Forest plot of Modified Ashworth Scale.

Two studies (30, 33) used the Modified Ashworth Scale to assess changes in spasticity before and after treatment (Figure 6). Meta-analysis showed that the overall mean difference was [MD = −0.34, 95% CI (−0.48, −0.20), *P* < 0.05]. Compared with the rTMS group or conventional treatment group, the MAS scores in the combined group were significantly reduced [MD = −0.27, 95% CI (−0.44, −0.09), *P* < 0.05, *I*^2^ = 39%]; [MD = −0.39, 95% CI (−0.44, −0.34), *P* < 0.05, *I*^2^ = 0%].

**Figure 7:**
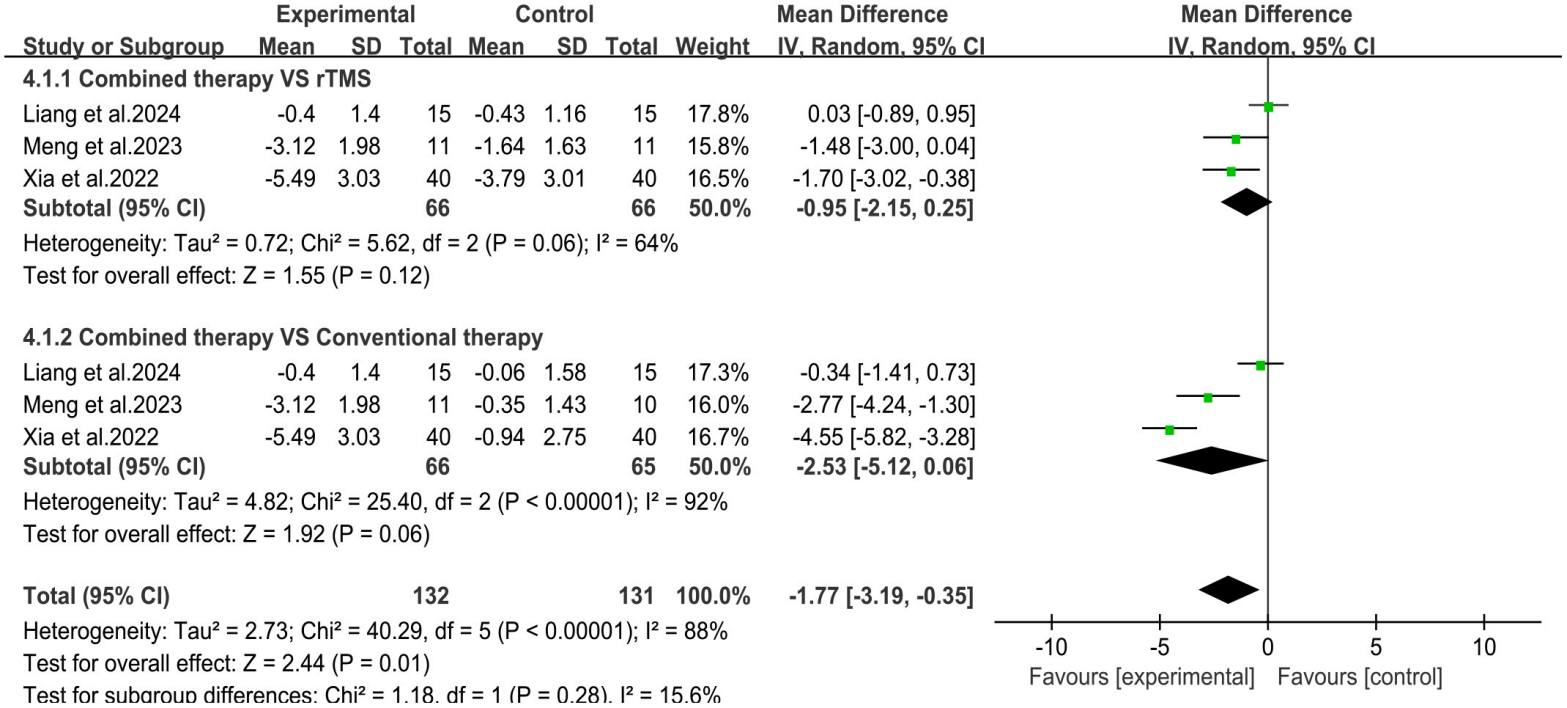
Forest plot of MEP latency.

**Figure 8:**
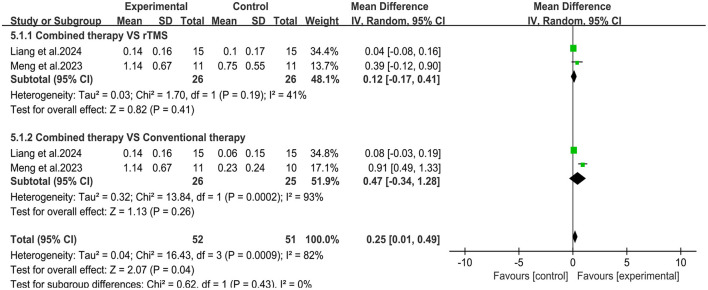
Forest plot of MEP amplitude.

Three studies (29, 33, 36) evaluated changes in motor evoked potential latency (MEP) latency (Figure 7). Meta-analysis showed that the overall mean difference in MEP latency was MD= −1.77, 95% CI (−3.19, −0.35), *P* < 0.05. However, no significant difference was obtained between the subgroups. Two studies (29, 36) reported changes in MEP amplitude (Figure 8). Meta-analysis showed that the overall mean difference in MEP amplitude was MD = 0.25, 95% CI (0.01, 0.49), *P* < 0.05. No significant difference was obtained between the subgroups.

### 3.5 Subgroup analysis of different transcranial magnetic stimulation protocols

**Figure 9:**
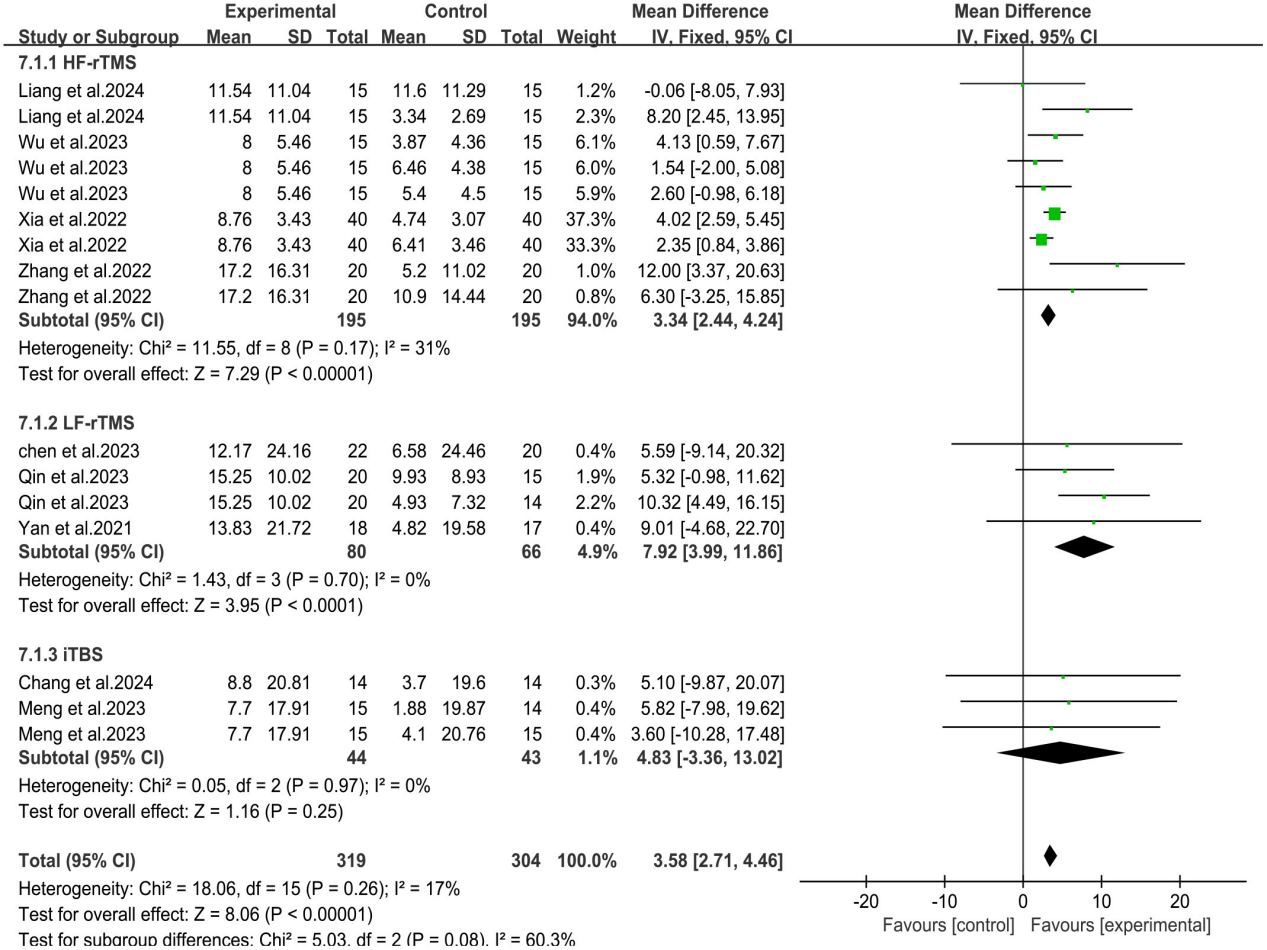
Forest plot of subgroup analysis for Fugl-Meyer Assessment-Upper Extremity.

**Figure 10:**
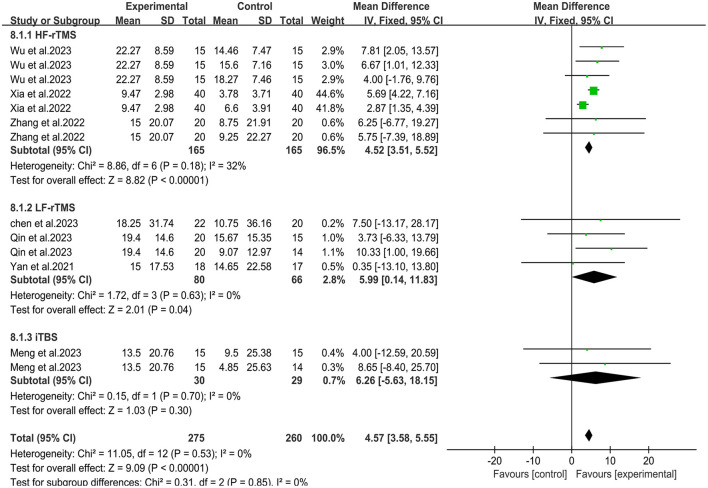
Forest plot of subgroup analysis for Modified Barthel Index.

To assess whether different transcranial magnetic stimulation (TMS) protocols influence the combined efficacy differently, we conducted a subgroup analysis based on these rTMS protocols. The subgroup analysis revealed that when HF-rTMS and LF-rTMS were employed as central interventions, the combination therapy significantly improved upper limb motor function in stroke patients compared to the control group [MD = 3.34, 95% CI (2.44, 4.24), *P* < 0.05; MD = 7.92, 95% CI (3.99, 11.86), *P* < 0.05] (Figure 9). However, there was no significant difference in clinical outcomes between the combined group receiving iTBS and the control group [MD = 4.83, 95% CI (−3.36, 13.02), *P* = 0.25]. Regarding the ability of daily living, The combination group of patients using HF-rTMS and LF-rTMS as central interventions showed significant improvement in their daily living activities [MD = 4.52, 95% CI (3.51, 5.52), *P* < 0.05; MD = 5.99, 95% CI (0.14, 11.83), *P* < 0.05] (Figure 10). However, similar to results of motor function, the clinical outcomes of the combined group receiving iTBS showed no statistically significant difference from the control group [MD = 6.26, 95% CI (−5.63, 18.15), *P* = 0.3].

### 3.6 Quality of evidence evaluation

According to the GRADE method, the level of evidence for the FMA-UE and MBI assessment group was low. For secondary outcomes, the GRADE assessment showed a low quality of evidence for MEP latency and MEP amplitude, and a very low quality of evidence for MAS. See Supplementary material for more details.

### 3.7 Meta-regression analysis

A meta-regression analysis of clinical characteristics (gender, age) was conducted. The findings indicate that neither the gender nor the age of the subjects has a statistically significant effect on the effect size of the meta-analysis (*p* = 0.502, *p* = 0.333). More details of the meta-regression analysis are shown in Supplementary material.

### 3.8 Sensitivity analysis and publication bias

The stability of the meta-analysis results was evaluated through sensitivity analysis, and the result of sensitivity analysis showed that our meta-analysis results were relatively stable. The results of Egger's test indicated no significant publication bias in the included studies. Specific details of the results of the sensitivity analysis and Egger's test are shown in Supplementary material.

## 4 Discussion

To evaluate the effectiveness of combined rTMS and rPMS on upper limb motor function recovery in stroke patients, we conducted this meta-analysis. This study reviewednine articles involving 483 patients. Our findings indicate that the therapy of LF-rTMS or HF-rTMS combined with rPMS is more effective than conventional therapy or rTMS alone in promoting the recovery of upper limb motor function and activities of daily living in post-stroke patients. In terms of neurophysiological indicators, meta-analysis showed that the MEP latency and amplitude were improved, but no significant differences were obtained between subgroups, and there was significant heterogeneity in the results.

A classic central-peripheral combined stimulation model is paired associative stimulation (PAS), a specific stimulation form of combined peripheral electrical stimulation (usually median nerve stimulation) and central TMS (37). Similar to our conclusions, Baroni et al.'s review (38) of the current literature on PAS in post-stroke motor rehabilitation indicated that PAS appears to have a role in stroke recovery. However, due to the limited number of studies and their heterogeneity, further evidence is needed to conclusively determine the effectiveness of PAS in stroke rehabilitation. Research has shown that the effect of PAS may be related to excitatory changes in cerebral cortex (39). Based on the principle of spike timing-dependent plasticity (STDP) (40), the effects of PAS are time-dependent, that is, the order of the two stimulations and the interstimulus interval (ISI) are key parameters determining the direction of excitability changes induced by PAS (37, 41). By adjusting the ISI, different effects on cortical excitability can be achieved, such as long-term potentiation (LTP) or long-term depression (LTD) in the motor cortex (37, 41). For instance, Stefan et al. (37) found that when the ISI was 25 ms (PAS25), with peripheral nerve stimulation followed by TMS, LTP-like effects were induced, leading to increased excitability in the primary motor cortex (M1). However, high-frequency electrical stimulation may cause discomfort during the treatment (42), which may limit the widespread application of this technology. Another central-peripheral combined stimulation model is transcranial direct current stimulation (tDCS) combined with peripheral electrical stimulation (PES). Research has shown that tDCS combined with PES can induce LTP- or LTD-like plasticity in the cortical areas of healthy individuals, and this effect is primarily dependent on the polarity of the tDCS (43). However, studies investigating this combined stimulation in stroke rehabilitation are limited, and the therapeutic outcomes reported across different studies are inconsistent (44–46), making it difficult to draw definitive conclusions about its efficacy. The combined rTMS and rPMS stimulation reported in this review can be regarded as a methodological improvement of the combined central-peripheral stimulation technique described above. Compared to peripheral electrical stimulation, rPMS can painlessly stimulate deeper muscle tissue that are inaccessible to electrical stimulation (47–49), and it does not require the patient to remove clothing during treatment. In addition, compared to the classical PAS mode, rPMS offers more flexibility in selecting peripheral stimulation points, such as nerve roots, nerve plexuses, or paralyzed muscle groups (22). Consistent with our findings, rTMS combined with rPMS has showed promising application in other patients after stroke. For example, in Yang et al.'s study (50), The authors observed positive effects of rTMS combined with rPMS on stroke patients with arm paralysis following contralateral seventh cervical nerve transfer (CSCNTS).

Although the combined application of rPMS and rTMS appears to show some promise in the rehabilitation of motor function after stroke, the exact mechanisms of the two forms of stimulation remain unclear. The application of rTMS is primarily based on the concept of interhemispheric competition (51). According to this concept, rTMS modulates cortical activity by applying excitatory or inhibitory stimulation to specific brain regions, thereby promoting a balance of excitability between the hemispheres and inducing neuroplastic changes (10, 11). For instance, LF-rTMS has been shown to significantly reduce motor evoked potential (MEP) amplitude in the contralesional primary motor cortex (M1) (52), while HF-rTMS enhances excitability in the ipsilesional M1 (53). The effectiveness and safety of rTMS in the treatment of upper limb motor function after stroke are well-documented (12, 17–21), and it has been more and more widely applied in clinic. While rTMS targeting the affected or unaffected hemisphere is a common clinical treatment for post-stroke, magnetic stimulation therapy targeting peripheral nerves and muscles has often been overlooked for a long time. Similar to peripheral neuromuscular electrical stimulation, rPMS applies a magnetic field at certain frequencies and intensities to peripheral nerves or muscles via Specific coils (54), and has been used to alleviate pain, reduce post-stroke spasticity, and promote motor recovery (13, 15, 49, 55, 56). The possible mechanism of rPMS is to stimulate peripheral nerves and muscles, increase the proprioceptive input from peripheral limbs to the central nervous system, and thus regulate the excitability of specific motor cortex and activate the reorganization process of central nervous system (14, 15, 57, 58). For example, Beaulieu et al. (14) found that PMS can enhance plasticity in the M1 and improve sensorimotor function in patients with chronic stroke, and this improvement may be generated through a large amount of “pure” proprioceptive input. In a study using positron emission tomography (PET) (15), the authors observed that the improvements in motor performance and spasticity in stroke patients following rPMS treatment were associated with significantly increased neural activation in the superior posterior parietal lobe and the premotor cortex (PM) areas.

Currently, the number of studies investigating the combined effects of rTMS and rPMS in improving upper limb motor impairment following stroke are limited, and the mechanisms underlying the combined therapeutic efficacy remain unclear. Previous research have shown that cortical reorganization may be one of the mechanisms underlying the recovery of motor function after stroke (15). Coincidentally, Stefan et al. (37) found that PAS can induce long-lasting cortical plasticity changes. Besides, in a study using the combination of rPMS and rTMS, Kumru et al. (42) found that the combination therapy induced an increase in MEP amplitude and a decrease in intracortical-inhibitory activity in the corresponding brain region, and that such changes were associated with combined central and peripheral stimulation. Therefore, we speculate that this neuroplasticity changes may be one of the underlying mechanisms for the combined effects observed in our study. Additionally, disruption of sensorimotor integration is prevalent in poststroke patient (59). Sensorimotor integration is the ability to process sensory input from the environment and integrate it with motor output to regulate movement (60), which plays a critical role in post-stroke motor learning (59, 61). However, in the study of the application of non-invasive neuroregulation techniques in the rehabilitation of motor function after stroke, most of the focus seems to have been on the direct regulation of neural activity in the motor cortex of the brain (62). The contribution of peripheral sensory feedback to motor control is often overlooked (59). Research has suggested that insufficient proprioceptive input may hinder post-stroke motor recovery (15). Therefore, increasing proprioceptive input from ascending sensory pathways may play an important role in motor recovery. Repetitive peripheral magnetic stimulation can enhance proprioceptive input to M1 from the limbs, facilitating motor output regulation and promoting sensorimotor integration (59). This sensorimotor integration fits with the rehabilitation concept proposed in recent studies (61, 63), that is, the central-peripheral closed-loop rehabilitation concept, an organic combination and synergistic therapy of top-down and bottom-up rehabilitation techniques, which can form a complete rehabilitation treatment loop and enhance the efficacy of a single intervention method. According to this concept, the combination of rTMS and rPMS, as a combination of central and peripheral therapies, is more effective in improving upper limb motor function after stroke than either therapy alone. The results of our study are consistent with this theory. As a non-invasive brain stimulation technique, rTMS promotes the restoration of bilateral cortical excitation balance and induces neuroplasticity changes by applying excitatory or inhibitory stimulation to specific regions of the brain (10, 11). Repetitive transcranial magnetic stimulation directly targets the cerebral hemisphere, providing top-down regulation of cortical activity, while repetitive peripheral magnetic stimulation targets peripheral tissue, providing bottom-up motor and sensory inputs to the cortex, thus completing a closed loop of magnetic stimulation (61).

It is worth noting that among the studies included in our analysis, only one study (29) utilized a paired repetitive magnetic stimulation protocol, in which the order of the two stimulations and the interstimulus interval (ISI) were clearly defined—factors that are critical in determining the type of PAS-induced cortical excitability modulation. Four studies (28, 30, 31, 34) employed unpaired magnetic stimulation, specifying only the sequence of the two interventions (rTMS first, followed by rPMS or vice versa) without providing specific ISI. The remaining four studies (32, 33, 35, 36) did not report the order or ISI between rPMS and rTMS. Unfortunately, none of the included studies explored the potential effects of the order and ISI of rPMS and rTMS in the intervention protocols. Consequently, it remains unclear how these factors may impact the combined therapeutic efficacy. Therefore, it is necessary to further investigate the sequential effects and ISI of rPMS and rTMS in future research to determine whether there are differences due to the different application sequences and ISI.

In addition, there was no standardized protocol for the optimal rTMS treatment parameters in the included studies. The rTMS parameters varied across studies included in our study, including LF-rTMS on the unaffected hemisphere, HF-rTMS or iTBS on the affected hemisphere, which might contribute to differences in therapeutic efficacy. Therefore, we conducted a subgroup analysis according to the various rTMS protocols. The results showed that the combined groups utilizing HF-rTMS and LF-rTMS as central interventions achieved significant improvements in Fugl-Meyer Assessment for Upper Extremity and Modified Barthel Index, whereas the clinical outcomes of the combined group utilizing iTBS showed no statistically significant difference from the control group. The results of subgroup analysis indicates that different rTMS protocols may yield varying impacts on the combined efficacy. However, it must be noted that subgroup analysis may compromise the randomization principle of the study, which may lead to a decrease in the accuracy of the results of the subgroup analysis. Therefore, we suggest that further research should consider evaluating the potential impact of different rTMS protocols combined with rPMS on improving functional impairment.

Our analysis has several limitations. Firstly, the number and sample size of studies included in this study are relatively small, and due to the deficiencies of randomization process and the missing of outcome data, there is a non-negligible risk of bias in the overall quality of the included studies, thus, our conclusions should be interpreted with caution. Secondly, most of the randomized controlled trials included in our study were conducted in Chinese mainland, which may limit the universal applicability of our research findings in other populations. Due to potential biases caused by geography and population, studies in western regions and populations are needed to confirm the efficacy of the combination therapy reported in this study. Additionally, based on the GRADE method for evidence quality assessment, most of the evidence quality ratings in this study were classified as “low,” with some rated as “very low.” Therefore, more high-quality evidence are needed to support our conclusion in the future. Finally, although this study carefully constructed search strategies and searched the database, there remains a possibility that relevant literature meeting our inclusion criteria was overlooked.

## 5 Conclusion

To our knowledge, this meta-analysis is the first to examine the synergistic effect of combined rTMS and rPMS on upper limb motor function recovery after stroke. Our findings suggest that high or low frequency rTMS combined with rPMS can promote the recovery of upper limb motor function and ability of daily living.

## Data Availability

The original contributions presented in the study are included in the article/Supplementary material, further inquiries can be directed to the corresponding authors.
