# Supplementary material for: Effects of repetitive transcranial magnetic stimulation combined with repetitive peripheral magnetic stimulation on upper limb motor function after stroke: a systematic review and meta-analysis
Source: Front Neurol. 2024 Nov 12;15:1472837. doi: 10.3389/fneur.2024.1472837 (PMC11588637; doi:10.3389/fneur.2024.1472837)
Supplement: Supplementary file 1 [file Table_1.docx]

Supplementary Material

# 1. Summary of methodological quality of evidence according to GRADE.

| **Quality assessment** | | | | | | | **No of patients** | | **Effect** | | **Quality** | **Importance** |
| --- | --- | --- | --- | --- | --- | --- | --- | --- | --- | --- | --- | --- |
|  |  |  |  |  |  |  |  |  |  |  |  |  |
| **No of studies** | **Design** | **Risk of bias** | **Inconsistency** | **Indirectness** | **Imprecision** | **Other considerations** | **rTMS+rPMS** | **Control** | **Relative (95% CI)** | **Absolute** |  |  |
| **FMA-UE** | | | | | | | | | | | | |
| 9 | randomised trials | serious^1^ | not serious | not serious | serious^2^ | none | 304 | 289 | - | MD 3.65 higher (2.75 to 4.54 higher) | ⊕⊕OO LOW | - |
| **FMA-UE - Combined therapy VS rTMS** | | | | | | | | | | | | |
| 9 | randomised trials | serious^1^ | not serious | not serious | serious^2^ | none | 179 | 171 | - | MD 2.49 higher (1.19 to 3.8 higher) | ⊕⊕OO LOW | - |
| **FMA-UE - Combined therapy VS Conventional therapy** | | | | | | | | | | | | |
| 6 | randomised trials | serious^1^ | not serious | not serious | serious^2^ | none | 125 | 118 | - | MD 4.69 higher (3.45 to 5.93 higher) | ⊕⊕OO LOW | - |
| **MBI** | | | | | | | | | | | | |
| 7 | randomised trials | serious^1^ | not serious | not serious | serious^2^ | none | 260 | 245 | - | MD 4.5 higher (3.5 to 5.5 higher) | ⊕⊕OO LOW | - |
| **MBI - Combined therapy VS rTMS** | | | | | | | | | | | | |
| 7 | randomised trials | serious^1^ | not serious | not serious | serious^2^ | none | 150 | 142 | - | MD 2.99 higher (1.56 to 4.42 higher) | ⊕⊕OO LOW | - |
| **MBI - Combined therapy VS Conventional therapy** | | | | | | | | | | | | |
| 5 | randomised trials | serious^1^ | not serious | not serious | serious^2^ | none | 110 | 103 | - | MD 5.95 higher (4.55 to 7.34 higher) | ⊕⊕OO LOW | - |
| **MAS** | | | | | | | | | | | | |
| 2 | randomised trials | serious^1^ | Serious^3^ | not serious | serious^2^ | none | 120 | 109 | - | MD 0.34 lower (0.48 to 0.2 lower) | ⊕OOO VERY LOW | - |
| **MAS - Combined therapy VS rTMS** | | | | | | | | | | | | |
| 2 | randomised trials | serious^1^ | not serious | not serious | serious^2^ | none | 60 | 55 | - | MD 0.27 lower (0.44 to 0.09 lower) | ⊕⊕OO LOW | - |
| **MAS - Combined therapy VS Conventional therapy** | | | | | | | | | | | | |
| 2 | randomised trials | serious^1^ | not serious | not serious | serious^2^ | none | 60 | 54 | - | MD 0.39 lower (0.44 to 0.34 lower) | ⊕⊕OO LOW | - |
| **MEP latency** | | | | | | | | | | | | |
| 3 | randomised trials | not serious | serious^1^ | not serious | serious^2^ | none | 132 | 131 | - | MD 1.77 lower (3.19 to 0.35 lower) | ⊕⊕OO LOW | - |
| **MEP latency - Combined therapy VS rTMS** | | | | | | | | | | | | |
| 3 | randomised trials | not serious | serious^1^ | not serious | serious^2^ | none | 66 | 66 | - | MD 0.95 lower (2.15 lower to 0.25 higher) | ⊕⊕OO LOW | - |
| **MEP latency - Combined therapy VS Conventional therapy** | | | | | | | | | | | | |
| 3 | randomised trials | not serious | serious^1^ | not serious | serious^2^ | none | 66 | 65 |  | MD 2.53 lower (5.12 lower to 0.06 higher) | ⊕⊕OO LOW | - |
| **MEP amplitude** | | | | | | | | | | | | |
| 2 | randomised trials | not serious | serious^1^ | not serious | serious^2^ | none | 52 | 51 | -- | MD 0.25 higher (0.01 to 0.49 higher) | ⊕⊕OO LOW | - |
| **MEP amplitude - Combined therapy VS rTMS** | | | | | | | | | | | | |
| 2 | randomised trials | not serious | not serious | not serious | serious^2^ | none | 26 | 26 | - | MD 0.12 higher (0.17 lower to 0.41 higher) | ⊕⊕⊕O MODERATE | - |
| **MEP amplitude - Combined therapy VS Conventional therapy** | | | | | | | | | | | | |
| 2 | randomised trials | not serious | serious^1^ | not serious | serious^2^ | none | 26 | 25 | - | MD 0.47 higher (0.34 lower to 1.28 higher) | ⊕⊕OO LOW | - |

CI: confidence interval; MD: mean difference. ^1^ Downgraded 1 level for serious risk of bias.^2^ Downgrade 1 level for small sample size and wide confdence interval.^3^ Downgraded 1 level for serious inconsistency.

# 2. Search strategies for all databases.

|  |
| --- |

| **Search strategy for Pubmed** | |
| --- | --- |
| Results: 231 | |
| Searches | Search Terms |
| #1 | ("Stroke"[Mesh]) OR ((((((((((((((((((((((((((((((((((((((((((((Strokes[Title/Abstract]) OR (Cerebrovascular Accident[Title/Abstract])) OR (Cerebrovascular Accidents[Title/Abstract])) OR (CVA[Title/Abstract])) OR (CVAs[Title/Abstract])) OR (Cerebrovascular Apoplexy[Title/Abstract])) OR (Apoplexy, Cerebrovascular[Title/Abstract])) OR (vascular Accident, Brain[Title/Abstract])) OR (Brain Vascular Accident[Title/Abstract])) OR (Brain Vascular Accidents[Title/Abstract])) OR (Vascular Accidents, Brain[Title/Abstract])) OR (Cerebrovascular Stroke[Title/Abstract])) OR (Cerebrovascular Strokes[Title/Abstract])) OR (Stroke, Cerebrovascular[Title/Abstract])) OR (Strokes, Cerebrovascular[Title/Abstract])) OR (Apoplexy[Title/Abstract])) OR (Cerebral Stroke[Title/Abstract])) OR (Cerebral Strokes[Title/Abstract])) OR (Stroke, Cerebral[Title/Abstract])) OR (Strokes, Cerebral[Title/Abstract])) OR (Stroke, Acute[Title/Abstract])) OR (Acute Stroke[Title/Abstract])) OR (Acute Strokes[Title/Abstract])) OR (Strokes, Acute[Title/Abstract])) OR (Cerebrovascular Accident, Acute[Title/Abstract])) OR (Acute Cerebrovascular Accident[Title/Abstract])) OR (Acute Cerebrovascular Accidents[Title/Abstract])) OR (Cerebrovascular Accidents, Acute[Title/Abstract])) OR (Ischemic Strokes[Title/Abstract])) OR (Stroke, Ischemic[Title/Abstract])) OR (Ischemic Stroke[Title/Abstract])) OR (cerebral infarction[Title/Abstract])) OR (Brain Infarction[Title/Abstract])) OR (Hemorrhagic Stroke[Title/Abstract])) OR (Hemorrhagic Strokes[Title/Abstract])) OR (Stroke, Hemorrhagic[Title/Abstract])) OR (Intracerebral Hemorrhagic Stroke[Title/Abstract])) OR (Hemorrhagic Stroke, Intracerebral[Title/Abstract])) OR (Intracerebral Hemorrhagic Strokes[Title/Abstract])) OR (Stroke, Intracerebral Hemorrhagic[Title/Abstract])) OR (Intracerebral Hemorrhage Stroke[Title/Abstract])) OR (Hemorrhage Stroke, Intracerebral[Title/Abstract])) OR (Intracerebral Hemorrhage Strokes[Title/Abstract])) OR (Stroke, Intracerebral Hemorrhage[Title/Abstract])) |
| #2 | ("Upper Extremity"[Mesh]) OR ((((((((((((((Extremities, Upper[Title/Abstract]) OR (Upper Extremities[Title/Abstract])) OR (Membrum superius[Title/Abstract])) OR (Upper Limb[Title/Abstract])) OR (Limb, Upper[Title/Abstract])) OR (Limbs, Upper[Title/Abstract])) OR (Upper Limbs[Title/Abstract])) OR (Extremity, Upper[Title/Abstract])) OR (arm[Title/Abstract])) OR (arms[Title/Abstract])) OR (hand[Title/Abstract])) OR (hands[Title/Abstract])) OR (shoulder[Title/Abstract])) OR (shoulders[Title/Abstract])) |
| #3 | #1 AND #2 |
| #4 | ("Transcranial Magnetic Stimulation"[Mesh]) OR (((((((((((((((((((((((((Magnetic Stimulation, Transcranial[Title/Abstract]) OR (Magnetic Stimulations, Transcranial[Title/Abstract])) OR (Stimulation, Transcranial Magnetic[Title/Abstract])) OR (Stimulations, Transcranial Magnetic[Title/Abstract])) OR (Transcranial Magnetic Stimulations[Title/Abstract])) OR (Transcranial Magnetic Stimulation, Single Pulse[Title/Abstract])) OR (Transcranial Magnetic Stimulation, Paired Pulse[Title/Abstract])) OR (Transcranial Magnetic Stimulation, Repetitive[Title/Abstract])) OR (repetitive transcranial magnetic stimulation[Title/Abstract])) OR (TMS[Title/Abstract])) OR (rTMS[Title/Abstract])) OR (magnetic stimulation[Title/Abstract])) OR (high-frequency repetitive transcranial magnetic stimulation[Title/Abstract])) OR (HF-rTMS[Title/Abstract])) OR (low-frequency repetitive transcranial magnetic stimulation[Title/Abstract])) OR (LF-rTMS[Title/Abstract])) OR (continuous theta-burst transcranial magnetic stimulation[Title/Abstract])) OR (continuous theta burst transcranial magnetic stimulation[Title/Abstract])) OR (CTBS[Title/Abstract])) OR (intermittent theta-burst stimulation[Title/Abstract])) OR (TBS[Title/Abstract])) OR (iTBS[Title/Abstract])) OR (Theta burst stimulation[Title/Abstract])) OR (θ-burst stimulation[Title/Abstract])) OR (continuous theta burst stimulation[Title/Abstract])) |
| #5 | (((repetitive peripheral magnetic stimulation[Title/Abstract]) OR (peripheral magnetic stimulation[Title/Abstract])) OR (rPMS[Title/Abstract])) OR (PMS[Title/Abstract]) |
| #6 | #4 OR #5 |
| #7 | (((((((randomized controlled trial[Publication Type]) OR (random[Title/Abstract])) OR (random allocation[Title/Abstract])) OR (single blind[Title/Abstract])) OR (double blind[Title/Abstract])) OR (RCT[Title/Abstract])) OR (controlled clinical trials[Title/Abstract])) OR (randomized[Title/Abstract]) |
| #8 | #3 AND #6 AND #7 |

| **Search strategy for Cochrane library** | |
| --- | --- |
| Results: 99 | |
| Searches | Search Terms |
| #1 | MeSH descriptor: [Stroke] explode all trees |
| #2 | MeSH descriptor: [Stroke Rehabilitation] explode all trees |
| #3 | (Stroke OR Strokes OR Cerebrovascular Accident OR Cerebrovascular Accidents OR CVA OR CVAs OR Cerebrovascular Apoplexy OR Apoplexy, Cerebrovascula OR vascular Accident, Brain OR Brain Vascular Accident OR Brain Vascular Accidents OR Vascular Accidents, Brain OR Cerebrovascular Stroke OR Cerebrovascular Strokes OR Stroke, Cerebrovascular OR Strokes, Cerebrovascular OR Apoplexy OR Cerebral Stroke OR Cerebral Strokes OR Stroke, Cerebral OR Strokes, Cerebral OR Stroke, Acute OR Acute Stroke OR Acute Strokes OR Strokes, Acute OR Cerebrovascular Accident, Acute OR Acute Cerebrovascular Accident OR Acute Cerebrovascular Accidents OR Cerebrovascular Accidents, Acute OR Ischemic Strokes OR Stroke, Ischemic OR Ischemic Stroke OR cerebral infarction OR Brain Infarction OR Hemorrhagic Stroke OR Hemorrhagic Strokes OR Stroke, Hemorrhagic OR Intracerebral Hemorrhagic Stroke OR Hemorrhagic Stroke, Intracerebral OR Intracerebral Hemorrhagic Strokes OR Stroke, Intracerebral Hemorrhagic OR Intracerebral Hemorrhage Stroke OR Hemorrhage Stroke, Intracerebral OR Intracerebral Hemorrhage Strokes OR Stroke, Intracerebral Hemorrhage):ti,ab,kw in Trials (Word variations have been searched) |
| #4 | #1 OR #2 OR #3 |
| #5 | MeSH descriptor: [Upper Extremity] explode all trees |
| #6 | (Upper Extremity OR Extremities, Upper OR Upper Extremities OR Membrum superius OR Upper Limb OR Limb, Upper OR Limbs, Upper OR Upper Limbs OR Extremity, Upper OR Arm OR Arms OR Hand OR Hands OR Shoulder OR shoulders):ti,ab,kw in Trials (Word variations have been searched) |
| #7 | #5 OR #6 |
| #8 | MeSH descriptor: [Transcranial Magnetic Stimulation] explode all trees |
| #9 | (Transcranial Magnetic Stimulation OR Magnetic Stimulation, Transcranial OR Magnetic Stimulations, Transcranial OR Stimulation, Transcranial Magnetic OR Stimulations, Transcranial Magnetic OR Transcranial Magnetic Stimulations OR Transcranial Magnetic Stimulation, Single Pulse OR Transcranial Magnetic Stimulation, Paired Pulse OR Transcranial Magnetic Stimulation, Repetitive OR repetitive transcranial magnetic stimulation OR TMS OR rTMS OR magnetic stimulation OR high-frequency repetitive transcranial magnetic stimulation OR HF-rTMS OR low-frequency repetitive transcranial magnetic stimulation OR LF-rTMS OR continuous theta-burst transcranial magnetic stimulation OR continuous theta burst transcranial magnetic stimulation OR CTBS OR intermittent theta-burst stimulation OR TBS OR iTBS OR Theta burst stimulation OR θ-burst stimulation OR continuous theta burst stimulation):ti,ab,kw in Trials (Word variations have been searched) |
| #10 | #8 OR #9 |
| #11 | (repetitive peripheral magnetic stimulation OR peripheral magnetic stimulation OR rPMS OR PMS):ti,ab,kw in Trials (Word variations have been searched) |
| #12 | #4 AND #7 AND #10 AND #11 |

| **Search strategy for Web of Science** | |
| --- | --- |
| Results: 22 | |
| Searches | Search Terms |
| #1 | (TS=(Stroke) OR AB=(Strokes OR Cerebrovascular Accident OR Cerebrovascular Accidents OR CVA OR CVAs OR Cerebrovascular Apoplexy OR Apoplexy, Cerebrovascular OR vascular Accident, Brain OR Brain Vascular Accident OR Brain Vascular Accidents OR Vascular Accidents, Brain OR Cerebrovascular Stroke OR Cerebrovascular Strokes OR Stroke, Cerebrovascular OR Strokes, Cerebrovascular OR Apoplexy OR Cerebral Stroke OR Cerebral Strokes OR Stroke, Cerebral OR Strokes, Cerebral OR Stroke, Acute OR Acute Stroke OR Acute Strokes OR Strokes, Acute OR Cerebrovascular Accident, Acute OR Acute Cerebrovascular Accident OR Acute Cerebrovascular Accidents OR Cerebrovascular Accidents, Acute OR Ischemic Strokes OR Stroke, Ischemic OR Ischemic Stroke OR cerebral infarction OR Brain Infarction OR Hemorrhagic Stroke OR Hemorrhagic Strokes OR Stroke, Hemorrhagic OR Intracerebral Hemorrhagic Stroke OR Hemorrhagic Stroke, Intracerebral OR Intracerebral Hemorrhagic Strokes OR Stroke, Intracerebral Hemorrhagic OR Intracerebral Hemorrhage Stroke OR Hemorrhage Stroke, Intracerebral OR Intracerebral Hemorrhage Strokes OR Stroke, Intracerebral Hemorrhage)) AND (TS=(Upper Extremity) OR AB=(Extremities, Upper OR Upper Extremities OR Membrum superius OR Upper Limb OR Limb, Upper OR Limbs, Upper OR Upper Limbs OR Extremity, Upper OR arm OR arms OR hand OR hands OR shoulder OR shoulders)) AND (TS=(Transcranial Magnetic Stimulation) OR AB=(Magnetic Stimulation, Transcranial OR Magnetic Stimulations, Transcranial OR Stimulation, Transcranial Magnetic OR Stimulations, Transcranial Magnetic OR Transcranial Magnetic Stimulations OR Transcranial Magnetic Stimulation, Single Pulse OR Transcranial Magnetic Stimulation, Paired Pulse OR Transcranial Magnetic Stimulation, Repetitive OR repetitive transcranial magnetic stimulation OR TMS OR rTMS OR magnetic stimulation OR high-frequency repetitive transcranial magnetic stimulation OR HF-rTMS OR low-frequency repetitive transcranial magnetic stimulation OR LF-rTMS OR continuous theta-burst transcranial magnetic stimulation OR continuous theta burst transcranial magnetic stimulation OR CTBS OR intermittent theta-burst stimulation OR TBS OR iTBS OR Theta burst stimulation OR θ-burst stimulation OR continuous theta burst stimulation)) AND (AB=(repetitive peripheral magnetic stimulation OR peripheral magnetic stimulation OR rPMS OR PMS)) AND (AB=(randomized controlled trial OR random OR random allocation OR single blind OR double blind OR RCT OR controlled clinical trials OR randomized)) |

| **Search strategy for Embase** | |
| --- | --- |
| Results: 262 | |
| Searches | Search Terms |
| #1 | 'cerebrovascular accident'/exp |
| #2 | 'stroke rehabilitation'/exp |
| #3 | strokes:ab,ti OR 'cerebrovascular accident':ab,ti OR 'cerebrovascular accidents':ab,ti OR cva:ab,ti OR cvas:ab,ti OR 'cerebrovascular apoplexy':ab,ti OR 'apoplexy, cerebrovascular':ab,ti OR 'vascular accident, brain':ab,ti OR 'brain vascular accident':ab,ti OR 'brain vascular accidents':ab,ti OR 'vascular accidents, brain':ab,ti OR 'cerebrovascular stroke':ab,ti OR 'cerebrovascular strokes':ab,ti OR 'stroke, cerebrovascular':ab,ti OR 'strokes, cerebrovascular':ab,ti OR apoplexy:ab,ti OR 'cerebral stroke':ab,ti OR 'cerebral strokes':ab,ti OR 'stroke, cerebral':ab,ti OR 'strokes, cerebral':ab,ti OR 'stroke, acute':ab,ti OR 'acute stroke':ab,ti OR 'acute strokes':ab,ti OR 'strokes, acute':ab,ti OR 'cerebrovascular accident, acute':ab,ti OR 'acute cerebrovascular accident':ab,ti OR 'acute cerebrovascular accidents':ab,ti OR 'cerebrovascular accidents, acute':ab,ti OR 'ischemic strokes':ab,ti OR 'stroke, ischemic':ab,ti OR 'ischemic stroke':ab,ti OR 'cerebral infarction':ab,ti OR 'brain infarction':ab,ti OR 'hemorrhagic stroke':ab,ti OR 'hemorrhagic strokes':ab,ti OR 'stroke, hemorrhagic':ab,ti OR 'intracerebral hemorrhagic stroke':ab,ti OR 'hemorrhagic stroke, intracerebral':ab,ti OR 'intracerebral hemorrhagic strokes':ab,ti OR 'stroke, intracerebral hemorrhagic':ab,ti OR 'intracerebral hemorrhage stroke':ab,ti OR 'hemorrhage stroke, intracerebral':ab,ti OR 'intracerebral hemorrhage strokes':ab,ti OR 'stroke, intracerebral hemorrhage':ab,ti |
| #4 | #1 OR #2 OR #3 |
| #5 | 'upper limb'/exp |
| #6 | 'upper extremity':ab,ti OR 'extremities, upper':ab,ti OR 'upper extremities':ab,ti OR 'membrum superius':ab,ti OR 'upper limb':ab,ti OR 'limb, upper':ab,ti OR 'limbs, upper':ab,ti OR 'upper limbs':ab,ti OR 'extremity, upper':ab,ti OR arm:ab,ti OR arms:ab,ti OR hand:ab,ti OR hands:ab,ti OR shoulder:ab,ti OR shoulders:ab,ti |
| #7 | #5 OR #6 |
| #8 | #4 AND #7 |
| #9 | 'transcranial magnetic stimulation'/exp |
| #10 | 'repetitive transcranial magnetic stimulation'/exp |
| #11 | 'transcranial magnetic stimulation':ab,ti OR 'magnetic stimulation, transcranial':ab,ti OR 'magnetic stimulations, transcranial':ab,ti OR 'stimulation, transcranial magnetic':ab,ti OR 'stimulations, transcranial magnetic':ab,ti OR 'transcranial magnetic stimulations':ab,ti OR 'transcranial magnetic stimulation, single pulse':ab,ti OR 'transcranial magnetic stimulation, paired pulse':ab,ti OR 'transcranial magnetic stimulation, repetitive':ab,ti OR 'repetitive transcranial magnetic stimulation':ab,ti OR tms:ab,ti OR rtms:ab,ti OR 'magnetic stimulation':ab,ti OR 'high-frequency repetitive transcranial magnetic stimulation':ab,ti OR 'hf rtms':ab,ti OR 'low-frequency repetitive transcranial magnetic stimulation':ab,ti OR 'lf rtms':ab,ti OR 'continuous theta-burst transcranial magnetic stimulation':ab,ti OR 'continuous theta burst transcranial magnetic stimulation':ab,ti OR ctbs:ab,ti OR 'intermittent theta-burst stimulation':ab,ti OR tbs:ab,ti OR itbs:ab,ti OR 'theta burst stimulation':ab,ti OR 'θ-burst stimulation':ab,ti OR 'continuous theta burst stimulation':ab,ti |
| #12 | 'repetitive peripheral magnetic stimulation'/exp |
| #13 | 'repetitive peripheral magnetic stimulation':ab,ti OR 'peripheral magnetic stimulation':ab,ti OR rpms:ab,ti OR pms:ab,ti |
| #14 | #9 OR #10 OR #11 OR #12 OR #13 |
| #15 | 'randomized controlled trial'/exp |
| #16 | #8 AND #14 AND #15 |

| **Search strategy for CNKI** | |
| --- | --- |
| Results: 38 | |
| Searches | Search Terms |
| #1 | （篇关摘：卒中 + 中风 + 脑卒中 + 脑中风 + 缺血性脑卒中 + 出血性脑卒中 + 脑血管意外 + 脑梗死 + 脑出血 + 脑血管病变 + 脑梗塞）AND（篇关摘：经颅磁刺激 + TMS + rTMS + 重复经颅磁刺激 + 磁刺激 + 中枢磁刺激）AND（篇关摘：外周磁刺激 + PMS + rPMS + 重复外周磁刺激） |

| **Search strategy for VIP** | |
| --- | --- |
| Results: 38 | |
| Searches | Search Terms |
| #1 | [((((((((((((题名或关键词=卒中 OR 题名或关键词=中风) OR 题名或关键词=脑卒中) OR 题名或关键词=脑中风) OR 题名或关键词=缺血性脑卒中) OR 题名或关键词=出血性脑卒中) OR 题名或关键词=脑血管意外) OR 题名或关键词=脑梗死) OR 题名或关键词=脑出血) OR 题名或关键词=脑血管病变) OR 题名或关键词=脑梗塞) AND (((((题名或关键词=经颅磁刺激 OR 题名或关键词=TMS) OR 题名或关键词=rTMS) OR 题名或关键词=重复经颅磁刺激) OR 题名或关键词=磁刺激) OR 题名或关键词=中枢磁刺激)) AND (((题名或关键词=外周磁刺激 OR 题名或关键词=PMS) OR 题名或关键词=rPMS) OR 题名或关键词=重复外周磁刺激))](https://qikan.cqvip.com/Qikan/search/index?LngMySearHistoryIdGuid=1761546c-138f-4134-a2e3-367f2c4c4f38&from=Qikan_Article_History) |

| **Search strategy for WanFang** | |
| --- | --- |
| Results: 39 | |
| Searches | Search Terms |
| #1 | (主题:(卒中) or 题名或关键词:(卒中 or 中风 or 脑卒中 or 脑中风 or 缺血性脑卒中 or 出血性脑卒中 or 脑血管意外 or 脑梗死 or 脑出血 or 脑血管病变 or 脑梗塞)) and (主题:(经颅磁刺激) or 题名或关键词:(经颅磁刺激 or TMS or rTMS or 重复经颅磁刺激 or 磁刺激 or 中枢磁刺激)) and（题名或关键词:(外周磁刺激 or PMS or rPMS or 重复外周磁刺激)） |

| **Search strategy for CMB** | |
| --- | --- |
| Results: 26 | |
| Searche | Search Terms |
| #1 | ("外周磁刺激"[常用字段:智能] OR "PMS"[常用字段:智能] OR "rPMS"[常用字段:智能] OR "重复外周磁刺激"[常用字段:智能]) AND ("经颅磁刺激"[常用字段:智能] OR "TMS"[常用字段:智能] OR "rTMS"[常用字段:智能] OR "重复经颅磁刺激"[常用字段:智能] OR "磁刺激"[常用字段:智能] OR "中枢磁刺激"[常用字段:智能]) AND ("卒中"[常用字段:智能] OR "中风"[常用字段:智能] OR "脑卒中"[常用字段:智能] OR "脑中风"[常用字段:智能] OR "缺血性脑卒中"[常用字段:智能] OR "出血性脑卒中"[常用字段:智能] OR "脑血管意外"[常用字段:智能] OR "脑梗死"[常用字段:智能] OR "脑出血"[常用字段:智能]) |

# 3. The results of the meta-regression analysis.


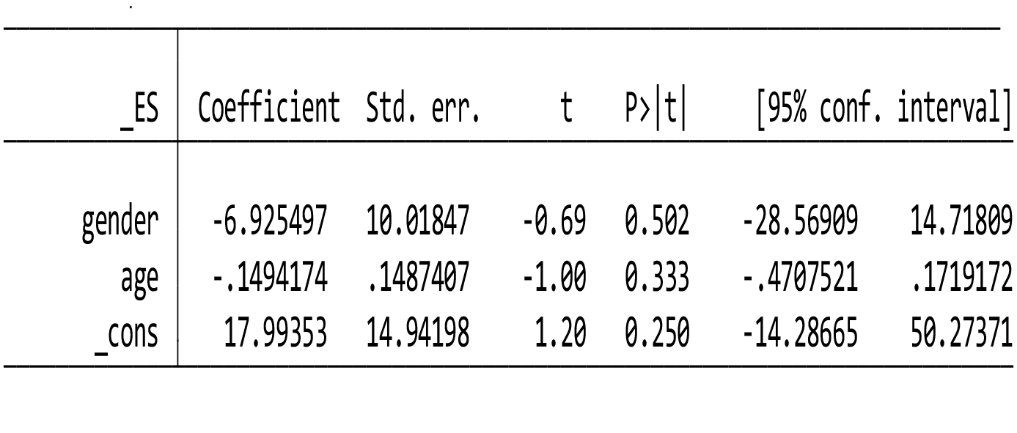


# 4. The results of the sensitivity analysis.


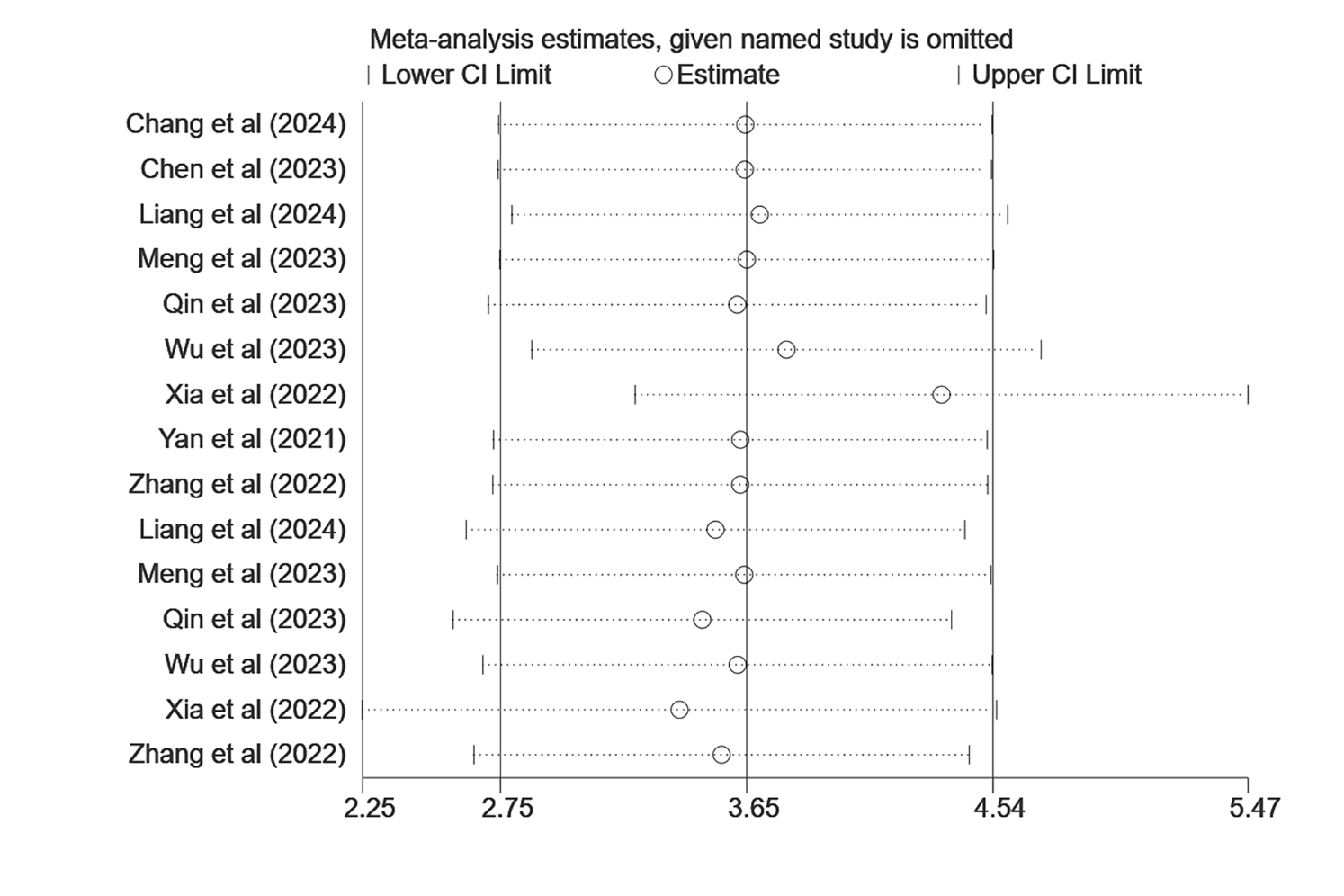


# 5. The results of the Egger's test.

| 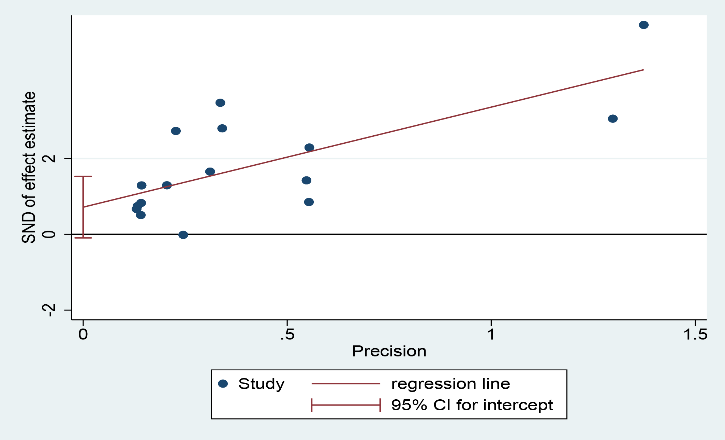 | 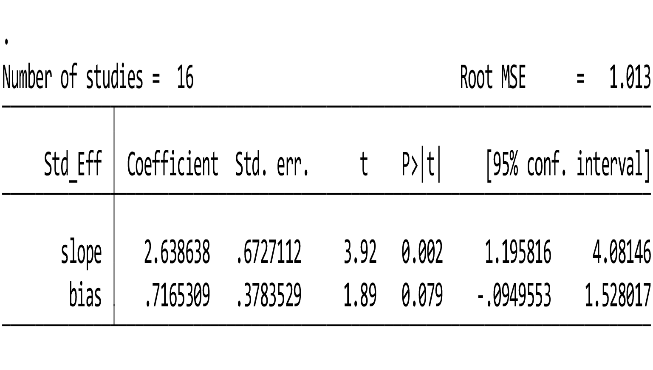 |
| --- | --- |
